# Author Correction: Immunogenicity and reactogenicity of SARS-CoV-2 vaccines BNT162b2 and CoronaVac in healthy adolescents

**DOI:** 10.1038/s41467-022-32337-6

**Published:** 2022-08-15

**Authors:** Jaime S. Rosa Duque, Xiwei Wang, Daniel Leung, Samuel M. S. Cheng, Carolyn A. Cohen, Xiaofeng Mu, Asmaa Hachim, Yanmei Zhang, Sau Man Chan, Sara Chaothai, Kelvin K. H. Kwan, Karl C. K. Chan, John K. C. Li, Leo L. H. Luk, Leo C. H. Tsang, Wilfred H. S. Wong, Cheuk Hei Cheang, Timothy K. Hung, Jennifer H. Y. Lam, Gilbert T. Chua, Winnie W. Y. Tso, Patrick Ip, Masashi Mori, Niloufar Kavian, Wing Hang Leung, Sophie Valkenburg, Malik Peiris, Wenwei Tu, Yu Lung Lau

**Affiliations:** 1 Department of Paediatrics and Adolescent Medicine, The University of Hong Kong, Hong Kong, China; 2 School of Public Health, The University of Hong Kong, Hong Kong, China; 3 HKU-Pasteur Research Pole, School of Public Health, The University of Hong Kong, Hong Kong, China; 4 Sir William Dunn School of Pathology, University of Oxford, Oxford, UK; 5 State Key Laboratory of Brain and Cognitive Sciences, The University of Hong Kong, Hong Kong, China; 6 Research Institute for Bioresources and Biotechnology, Ishikawa Prefectural University, Nonoichi, Japan; 7 IRCCS, Humanitas Research Hospital, Milan, Italy; 8 Department of Microbiology and Immunology, Peter Doherty Institute for Infection and Immunity, University of Melbourne, Melbourne, VIC Australia; 9 Centre for Immunology and Infection, Hong Kong, China

**Keywords:** SARS-CoV-2, Inactivated vaccines, RNA vaccines

Correction to: *Nature Communications* 10.1038/s41467-022-31485-z, published online 28 June 2022

In this article, the affiliation ‘HKU-Pasteur Research Pole, School of Public Health, The University of Hong Kong, Hong Kong, China’ for Carolyn A. Cohen, Asmaa Hachim, Niloufar Kavian and Sophie Valkenburg was missing. The original article has been corrected.

